# Editorial: Mitochondrial Dysfunction and Cardiovascular Diseases

**DOI:** 10.3389/fcvm.2021.645986

**Published:** 2021-01-22

**Authors:** Junichi Sadoshima, Richard N. Kitsis, Sebastiano Sciarretta

**Affiliations:** ^1^Department of Cell Biology and Molecular Medicine, Rutgers New Jersey Medical School, Newark, NJ, United States; ^2^Albert Einstein College of Medicine, Bronx, NY, United States; ^3^Department of Medical and Surgical Sciences and Biotechnologies, Sapienza University of Rome, Latina, Italy; ^4^IRCCS Neuromed, Pozzilli, Italy

**Keywords:** mitochondrial dysfunction, mitophagy, cardiovascular diseases, mitochondrial dynamics, mitochondrial ROS

A deeper understanding of the molecular mechanisms underlying the development and progression of cardiovascular diseases represents a major goal in cardiovascular medicine. Mitochondrial dysfunction has emerged as major player in the development of cardiovascular diseases, with potential therapeutic implications. Mitochondrial dysfunction encompasses mitochondrial complex disruption, mitochondrial uncoupling, and cristae remodeling and swelling, which in turn cause ROS accumulation, energy stress, and cell death.

This Research Topic is a collection of original and state-of-the art review articles discussing and extending our current knowledge about molecular mechanisms responsible for mitochondrial dysfunction in cardiovascular diseases. Many aspects of mitochondrial biology and therapies targeting damaged mitochondria have been highlighted.

One of the main feature of mitochondrial dysfunction observed in several cardiovascular diseases is the exaggerated generation of mitochondrial ROS (1), which represents the common pathological substrate underlying diabetes-induced complications, such as cardiomyopathy, as comprehensively described by Kaludercic and Di Lisa in their review article. Mitochondrial ROS are generated from multiple sources in cardiomyocytes during diabetes by a feed-forward/amplification mechanism, which further exacerbates oxidative stress and causes contractile dysfunction. The authors reviewed current therapies aimed at reducing ROS and improving cardiac function in diabetic patients. While some systemic antioxidants failed to exert cardiac protection in clinical trials, mitochondrial-targeted antioxidants such as MitoTEMPO were shown to be cardioprotective in preclinical models of diabetic cardiomyopathy.

Sodium glucose cotransporter 2 (SGLT2) inhibitors also appear to be promising drugs to reduce cardiovascular events in diabetic patients. In this regard, Maejima provided a detailed overview about the mitochondrial-mediated mechanisms underlying the beneficial effects of SGLT2 inhibitors in heart failure. SGLT2 inhibitors increase ketone bodies, which represent a suitable source of energy in failing hearts, and also improve sodium metabolism and mitochondrial dynamics. However, further studies are needed to identify other targets modulated by SGLT2 inhibitors, since SGLT2 does not appear to be expressed in human and rodent cardiomyocytes, at least in unstressed conditions. A modulation of mitochondrial dynamics may contribute to the beneficial effects of this class of drugs on mitochondrial function in response to metabolic derangements (2).

Targeting mitochondria, and in particular mitochondrial ROS, has also emerged as a potential therapy for patients with dilated cardiomyopathy with ataxia syndrome (DCMA), a rare genetic disorder caused by a mutation of DNAJ Heat Shock Protein Family (Hsp40) Member C19 (DNAJC19), a protein localized in the inner mitochondrial membrane. Machiraju et al. demonstrated that SS-31, a mitochondrial targeted antioxidant, also known as elamipretide or Bendavia, rescues mitochondrial fragmentation, oxidative stress, and improves mitochondrial fusion in skin fibroblasts extracted from DCMA patients. However, the therapeutic potential of SS-31 in improving cardiac function in patients with DCMA should be assessed in further studies.

Mitochondrial health is facilitated by specific quality control mechanisms, such as mitophagy, a cargo-specific form of autophagy selective for elimination of damaged mitochondria (3). Damaged mitochondria are degraded by mitophagy and defects in mitophagy were reported to lead to harmful cardiovascular effects, because of accumulation of defective mitochondria. In their original article, Thomas et al. found decreased levels of Parkin protein in the heart of obese mice. Parkin is a ubiquitin E3 ligase, which represents a canonical regulator of mitophagy and proteasome degradation. The authors also observed a modest increase of infarct size in obese mice undergoing ischemia/reperfusion (IR) *ex-vivo* and a cardiac accumulation of ubiquitinated mitochondrial proteins at baseline and in response to IR in obese animals. This study suggested that mitophagy may be impaired in the context of obesity because of Parkin downregulation, thereby predisposing the heart to develop increased injury in response to stress. However, a direct assessment of mitophagy was not performed in this study and further work is necessary to clarify the impact of metabolic alterations on Parkin-dependent and independent mitophagy in the heart.

The importance of autophagy and mitophagy abnormalities in aging-induced cardiovascular abnormalities was the main focus of the review article by Liang and Gustafsson. The authors reviewed relevant literature supporting the concept that autophagy declines with aging, leading to age-related cardiovascular diseases, due to alterations in cellular energy metabolism and adaption to stress. Either genetic or pharmacological activation of mitophagy appears to attenuate aging-related abnormalities, whereas its inhibition seems to accelerate them (4). It will be important to understand in the future how aging affects Parkin-dependent and independent mitophagy in the heart, and the exact molecular mechanisms through which autophagosome formation and fusion are impaired by the aging process. Increased oxidative stress and inflammation appear to play a critical role.

Aside from mitophagy and mitochondrial dynamics, mitochondrial proteostasis is also emerging as an important mechanism regulating mitochondrial quality control in the heart, as described in the paper by Arrieta et al. Mitochondrial proteostasis regulates biogenesis, folding, and degradation of mitochondrial proteins and this process appears to be altered during cardiac stress. In the presence of misfolded protein accumulation in mitochondria, mitochondrial unfolded protein response (mtUPR) is activated by means of accumulation of ATF5, which translocates to the nucleus and stimulates the upregulation of an adaptive gene response aimed at the restoration of mitochondrial protein folding and proteostasis. Previous work showed that stimulation of mtUPR improves mitochondrial function and reduces cardiac damage in response to I/R injury and pressure overload. The elucidation of the integration points between mitochondrial and endoplasmic reticulum proteostasis represents an important aspect to be clarified in future studies.

Mitochondria are also massively damaged by anthracycline-based chemotherapy, and mitochondrial dysfunction contributes to the development anthracycline-induced cardiotoxicity, as reviewed by Murabito et al. Doxorubicin, a well-known drug belonging to the anthracycline class, directly binds cardiolipin and accumulates into mitochondria, causing disruption of electron transport chain complexes, thereby contributing to ROS accumulation. The latter triggers several adverse events, such as mitochondrial uncoupling, oxidative stress, apoptosis, ferroptosis, and impairment of calcium metabolism, which then lead to cardiomyopathy development. In addition, mitochondrial dynamics and autophagy are impaired by doxorubicin treatment, further aggravating mitochondrial damage. Different therapeutic strategies have been suggested to reduce anthracycline-induced mitochondrial dysfunction and cardiotoxicity. These include mitochondria-targeted antioxidants, autophagy activators, or inhibitors of mitochondrial fatty acid beta-oxidation. However, the signaling pathways involved in the perpetuation of mitochondrial damage in response to doxorubicin treatment still need to be clarified. The elucidation of this aspect will be very important for the discovery of new therapeutic targets for the prevention of doxorubicin-induced cardiotoxicity and for the identification of subjects with potentially higher susceptibility to develop cardiac injury after chemotherapy.

Mitochondrial biogenesis is also critical for the regulation of mitochondrial turnover and function in cardiovascular pathophysiology (5). The transcriptional coactivator peroxisome proliferator-activated receptor γ coactivator 1 alpha (PGC-1α) represents a major regulator of mitochondrial biogenesis and metabolism, as discussed in detail by Oka et al. A dysregulation of PGC-1α signaling during heart failure occurs at transcriptional and post-transcriptional level, contributing to the development of cardiac dysfunction, due to alterations of multiple mechanisms, particularly those involved in mitochondrial metabolism.

Perturbations of epigenetic mechanisms regulating mitochondrial function also contribute to cardiovascular diseases, as reviewed by Mohammed et al. Epigenetic changes impair mitochondrial function, resulting in a decrease in mitochondrial metabolites (i.e., NAD, FAD) used as cofactors by components involved in chromatin modifications. The latter further exacerbates epigenetic remodeling. Among epigenetic modulators, HDAC inhibitors or SIRT1-3 activators were shown to preserve mitochondrial function in different cardiovascular diseases by reducing epigenetic remodeling.

In conclusion, this Research Topic highlights that alterations in different mechanisms regulating mitochondrial quality control and function directly contribute to the development of cardiovascular diseases. Mitochondrial dysfunction determines an impairment of energy production, which is detrimental for heart function. In addition, mitochondrial damage triggers cell death pathways. Although the reduction of mitochondrial ROS appears to be a valid approach to reduce mitochondrial dysfunction, an improvement of mitochondrial quality control and epigenetic mechanisms may also represent an efficacious strategy in future clinical applications.

## Author Contributions

All authors listed have made a substantial, direct and intellectual contribution to the work, and approved it for publication.

## Conflict of Interest

The authors declare that the research was conducted in the absence of any commercial or financial relationships that could be construed as a potential conflict of interest.
